# Information manipulation and historical revisionism: Russian disinformation and foreign interference through manipulated history-based narratives

**DOI:** 10.12688/openreseurope.16087.1

**Published:** 2023-07-27

**Authors:** Cristina M. Arribas, Rubén Arcos, Manuel Gértrudix, Kamil Mikulski, Pablo Hernández-Escayola, Mihaela Teodor, Elena Novăcescu, Ileana Surdu, Valentin Stoian, Antonio García-Jiménez

**Affiliations:** 1 Universidad Rey Juan Carlos, Móstoles, Community of Madrid, 28933, Spain; 2 Maldita.es, Madrid, Spain; 3 Academia Nationala de Informatii Mihai Viteazul, Bucharest, Bucharest, Romania

**Keywords:** Disinformation, Historical Revisionism, Foreign Information Manipulation and Interference, Hybrid Threats, Narratives, Russian propaganda, pro-Kremlin disinformation, Resilience

## Abstract

**Background:** Disinformation and historical revisionism have been acknowledged as tools for foreign interference that belong to the landscape of hybrid threats. Historical revisionism plays an essential role in Russian foreign policy towards the post-Soviet space and is in strong relation with the concepts of Near Abroad and Russkii Mir (‘Russian World’) and with certain ideas contained in the neo-Eurasianist Movement. This article examines Russian revisionist narratives disseminated in information and influencing campaigns in Europe and against the West.

**Methods:** This study uses a mixed methodology combining desk research, including literature review, and analysis of the EUvsDisinfo database of cases identified before the February 2022 invasion of Ukraine.

**R** **esults:** The manipulation of historical events has been largely employed by the Kremlin as a tool for foreign interference to achieve strategic objectives. First World War treaties, mainly the Trianon Peace Treaty, as well as the Second World War and the communist and fascist historical experiences in countries within the post-Soviet space, are the pivotal topics from which hostile influencing narratives are built. From the analysis of the EUvsDisinfo database, the article identifies seven topic themes.

**Conclusions: ** Our findings suggest that pre-emptively elaborated counter-narratives based on historical evidence and sound historiography can be an effective tool against hostile revisionist narratives that exploit vulnerabilities and specific target groups within European societies.

## Introduction

The invasion of Ukraine on 24 February 2022 by the Russian Federation, preceded by the illegal annexation of Crimea and Sevastopol in 2014, requires a careful examination and reflection on the symbolic tools employed by the Kremlin to attempt to justify the military aggression and these actions to different audiences, (foreign and domestic). The Kremlin has a long history of engaging in the use of propaganda, disinformation, and information manipulations, but it is the strategic use of historical revisionism and hostile narratives based on manipulated history by the Kremlin (and its ecosystem of actives and affiliated organizations) what in a context of preparation of its military offensive emerges as a necessary and most relevant tool. Historical revisionism and manipulated history-based narratives have been employed to legitimize its imperialist foreign policy agenda and achieve its geopolitical goals. Similarly, within the broader framework of analysis, the concepts of ‘Near Abroad’ and ‘Ruskii Mir’ (Russian World), as well as the neo-Eurasianism ideology ( Pizzolo, 2020) on the creation of a Eurasian space as a counter-concept to the West ( Wittke, 2018) must be taken into consideration. 

On the other hand, it is well known that the collapse of the Soviet Union led to a necessary ‘moment’ or process of identity questioning/reformulation, or identity assertiveness, in the countries of the post-Soviet space. In this context, the revision of history emerged as a fundamental pillar from which to define new (and sometimes former) sovereign states ( Cheskin, 2012; Moll, 2013; Tolz, 1998), by focusing on facing/dealing with the 20th Century communist and fascist experiences ( Belavusau *et al.,* 2021; Braham & Hanebrink, 2020; Đureinović, 2018; Pavasović Trošt, 2018) and on territorial and ethnic issues rooted in the First World War peace treaties. This revision of history, based on the historiographical research and the sound work of historians, is precisely the most effective tool against the manipulative revision of historical events and its weaponization by the Kremlin. 

In Central and Eastern Europe (CEE) these processes culminated in a rapprochement to the West ( Kazharski, 2019) and, for most of the countries, ended up with their integration as members of the European Union (EU) and the North Atlantic Treaty Organization (NATO). For Russia, however, these processes, after Putin’s arrival, crystallized in an imperialist foreign policy, and agenda, that “conceptualize all other fourteen newly independent states—with some variation when it comes to the Baltic States—that formerly belonged to the Soviet Union as its Near Abroad (blizhnee zarubezh’e), a politicized geographic space where Russia has special interests and influence and that appears, in effect, to be a space of particular contested, conditional, and hierarchal sovereignties” ( Wittke, 2018, p. 3).

History rewriting became a state effort for the Russian Federation with a double purpose: promoting Russia’s own interpretation of historical events for leading “to the inclusion of the Soviet heritage both temporally and spatially,” and “limiting the influence of conflicting Western narratives inside the Russian public domain” ( Kratochvíl & Shakhanova, 2020, p. 1).

The employment of this manipulated memory (domestically and abroad) has been a salient feature of Russian political and media discourse that can be traced back to the Soviet period to legitimize the intervention in its space of influence ( Luxmoore, 2019).

This article examines historical revisionism-based information and influencing campaigns conducted by Russia’s structure of propaganda, focusing not only in exploring specific narratives, but also elucidating the historic events exploited, the audiences targeted, and actors aligned with the Kremlin within the European space that have contributed to their diffusion.

The article, framed under the EU-HYBNET project research activities, employs a mixed methodology that combines desk research, including literature review, and the analysis of databases of mis- and disinformation –specifically, the EUvsDisinfo database for the period 2016–2022– and reports from European institutions, think tanks, and fact-checking organizations.

### Historical revisionism, information manipulations and hybrid threats/warfare

The EU has deplored the use by Russia of “obvious lies, information manipulation and historical revisionism” to justify the war of aggression against Ukraine ( Delegation of the EU to the International Organizations in Vienna, 2023). The spread of disinformation and manipulative historical narratives have been acknowledged as tools for foreign interference that belong to the landscape of hybrid threats ( Cullen *et al.,* 2021, p. 9). Similarly, lack of awareness on disinformation and “poor knowledge of history” are societal vulnerabilities that have being exploited by foreign actors to influence and interfere ( Jungwirth *et al.*, 2023, p. 81).

Addressing historical revisionism requires a careful treatment ( Cattini, 2011) and taking into consideration terminology nuances. History must be understood as a “continuum dialogue between the present and the past” and the practices of revision are the “lifeblood of the historical scholarship” ( McPherson, 2003). History serves the collective experience of each generation. Societies and their mindsets are in a permanent mutation process, and these changes demand a revision of the previous values and preconceptions to bring coherence to the new reality. In this context, the revision of history has a lawful purpose. But misrepresenting history for leading to socio-political change and serving partisan political goals, including those in foreign and defence policy, is a bad praxis. In this sense, scholars establish a distinction between a legitimate revisionism, that is produced when “new evidence is available and new questions are asked” ( Grossman, 2019) and bad revisionism, in a pejorative sense when the history is manipulated for political ends with a complete lack of scientific foundation ( Cattini, 2011). In these cases, revisionism becomes a “synonym for not telling the truth” ( Grossman, 2019). Tucker (2008, p. 3) makes a distinction between revisionist historiography and revised historiography. The first one, “is uniquely founded on the penchant for therapeutic values over cognitive values” and “attempts to confuse knowledge with fiction are founded on bad philosophy, invalid arguments, and misunderstandings of contemporary epistemology and philosophy of science.” ( Tucker, 2008, p. 3). On the contrary, revised historiography is evidence-based revision that results when new items of evidence are discovered by historians; it is marked by the sound prevalence of cognitive values in the practice of historiography over other values in historiographic interpretations (Ibid.). As explained by Tucker,

“Frequently-used therapeutic values in historiography include the denial of historical guilt, for instance through denying the Holocaust; the promotion of self-respect, for instance via national myths; and the elimination of a sense of alienation and absurdity, for instance through conspiracy theories.” (2008, p. 5).

According to this frame, historical revisionism emerges as a manipulation tool at the service of authoritarianism, imperialist foreign policy goals, and the agendas in the domestic arena of political parties in the extremes of the ideological spectrum.

State efforts to (re)construct the history need narratives to conduct people towards a certain collective memory and to create a “normative vision of the future” ( Cianciara, 2021, p. 4)

According to Kaiser & McMahon (2017), narrative, as a concept, refers to “a series of events or developments told, more or less cohesively, along a storyline or plot” (p. 149) and highlights its character of political weapon and its “key role in the social construction of nations and in the political integration of states since the nineteenth century” (p. 150). They serve the goals of the “narrative entrepreneur” through the selection of historical accounts of the events and constitute a normative vision of a political project ( Cianciara, 2021, p. 4)

The intentional creation, shaping and propagation of narratives for (geo)political purposes is a fundamental aspect involved in information operations ( Wilson *et al.,* 2018). The practice of “active measures” by Russia’s intelligence organizations during the Soviet period is well known and currently most of these tactics of political warfare and covert influence abroad have been recognized as tools for hybrid warfare/threats. While in our current digital informational landscape, public diplomacy aims to persuade and/or gain credibility, online active measures seek to confuse and distort information spaces ( Pomerantsev & Weiss, 2014). The Kremlin´s information operations are developed within foreign countries to promote disaffection between domestic groups towards institutions, provoke favourable attitudes to the unacknowledged sponsor state and interfering in the democratic decision-making processes of targeted countries and societies; they have been largely employed as essential tools in the hybrid warfare conducted against Ukraine since the illegal annexation of Crimea and Sevastopol in 2014.

Historical revisionism plays an essential role in the Russian foreign policy towards the post-Soviet space and is in strong relation with the concepts of Near Abroad and Russkii Mir, and with certain ideas contained in neo-Eurasianist Movement. These constructions establish the basis for Russia’s space of interest and influence, combining different analytical approaches. ‘Near abroad’ can be defined as a politicized geographic space of “particular contested, conditional, and hierarchal sovereignties” ( Wittke, 2018, p. 3) that include all the countries that formed the Soviet Union. Hence, it works on a geographical level. ‘Russkii Mir’, on the other hand, functions on a cultural and historical level, joining Russian legacy inside and outside Russia. As pointed out by Pizzolo (2020, p. 7), elements of the neo-Eurasianist doctrine with regard to Putin’s regime can be found in aspects such us “the will to rediscover Russian Imperial identity, in the interest for integrating Eurasian regions, in the support for the advent of a multipolar world, in the consolidation among society of traditional values that contrast to Western cosmopolitism and globalism” ( Pizzolo, 2020, p. 7). In particular, the opposition to those Western liberal democratic values make pro-Russian or pro-Kremlin narratives attractive for national populist political parties within the European Union member countries.

## Methods

The article, framed under the EU-HYBNET project research activities, employs a mixed methodology that combines desk research, including literature review, and the analysis of databases of mis- and disinformation –specifically, the EUvsDisinfo database for the period 2016–2022– and reports from European institutions, think tanks, and fact-checking organizations.

### Desk research

We conducted desk research for obtaining a prior approximation to our topic of study and contextualizing the phenomena of historical revisionism and its potential application in Foreign Information Manipulation and Interference (FIMI) activities and in hybrid warfare. We identified scientific literature, and reports from think-tanks and other organizations with the following specific objectives:

1. Explore historical revisionism as a manipulative tool within the context of hybrid warfare/threats.
2. Describe the role of revision of history in the processes of national identity building within the post-soviet space.
3. Analyze the use of historical revisionism by state actors and illiberal political parties within the EU.
4. Examine the historical revisionism-based narratives disseminated by the Russian Federation and other actors.

As a retrieval strategy for gathering the most relevant recent literature, we defined general keywords to conduct searches; search equations were developed for performing combinations of general keywords with other specific ones:

“General word” AND (“Specific word 1” OR “Specific word 2” OR “Specific word 3” […]).

These search strategies can be consulted in the extended data ( Arribas *et al.,* 2023b).

We established as a time frame for the retrieval the period 2014–2021. This is premised on the fact that the illegal annexation of Crimea by the Russian Federation was operated in March 2014 and, hence, it was reasonable to expect an increase in the academic interest on the use of manipulation of history accompanying Russian propaganda and hostile narratives against Ukraine and the West. The searches were conducted in October 2021 in these major academic databases: Scopus, Web of Science (WoS), and the Central and Eastern European Online Library (CEEOL). The searches started with a preselection of 912 results (only articles were included). After filtering by keywords regarding the regional sphere of interest for the research, and eliminating the duplicated results, the sample was reduced to 116 documents. Finally, 31 were selected based on the criteria of relevance to the topic of historical revisionism.

In addition to searches in major academic databases, we gathered relevant findings from research papers and reports from governmental organizations and think-tanks and focused on the Baltics and Central and Eastern European (CEE) countries. The selection criteria was based on the assessed relevancy with hot historical revisionist topic themes.

### Analysis of the EUvsDisinfo database

An analysis of disinformation cases was also conducted to identify key historical events exploited, and strategies used in revisionist narratives. The EUvsDisinfo database (European External Action Service) was employed to extract the cases. By employing search terms related to 20 ^th^ Century major political events and themes, a total of 408 items were retrieved for the period 2016–2022. The earliest disinformation case was published in 2016 (the EUvsDisinfo database was set up in 2015) and the latest in January 10th, 2022. A structured analytic framework based on Lasswell’s communication construct which is based on answers provided for the questions “Who says what, on behalf of whom (or on its own behalf), with what intentions, in what situations, with what assets, using what strategies (key messages and channels), to which audiences, and producing what kind of effects?”( Arcos, 2018, p. 5) was is employed to analyze the results. According to Rubén Arcos, Lasswell’s model allows:

“A systematic examination of the communication process by answering the key questions posed by the model drives critical thinking in the consumption of information, provides exposure to the elements involved, and unveils the patterns and dynamics of persuasion through the use of communication (symbolic and behavioural). This is key for providing an understanding of the influencing attempts, and for developing strategies and tactics for counteracting the effects of influence operations.” ( Arcos, 2018, p. 5)

The search strategy used for the EUvsDisinfo database was aimed to identify the main narratives against EU and NATO. In this respect, we employed specific search terms such as ‘historical revisionism’, “historical revisionism WWII”, “historical revisionism WWII Poland”, “historical revisionism WEST”, “historical revisionism EU”, “historical revisionism NATO” and ‘Yugoslavia’. A total of 424 items were retrieved: 408 for all the versions of the term ‘historical revisionism’ and 16 for the term ‘Yugoslavia’. The latest term was proposed based on the assumption that it would be likely to find it in relation to NATO intervention and the Russian interpretations of the events. The earliest disinformation case was published in March 2016 and the latest in January 2022, the moment when the research was conducted.

## Results

### Desk research and analysis

We first present and discuss findings from desk research and analysis. Results are organized according to the following structure: historical events and narratives exploited, agency of Russian and pro-Russian amplifiers; intend, effects, and resilience.

***Historical events and narratives exploited.*** The First World War treaties that lead to the reshaping of the territorial borders and ethnic minorities distribution, together with some interwar events in the CEE space such as the Poland-Ukraine war (1918–1919), treaties preceding the Second World War, and the experience of a Communist and National Socialist past constitute the main revisionist topic themes exploited in information manipulation campaigns.

The Russian Federation “attaches great importance to the dissemination of a strategic narrative supportive of Russian foreign policy and its appeal stems from its ability to blend with already existing, frequently socially conservative, nationalistic and anti-Wester discourse among target audiences abroad” ( Nilsson, 2021, p. 63). Tyushka (2022) has shown how the narratives “became weaponized as part of Russia’s matryoshka-style multi-layered conflict in the simultaneous fight against Ukraine’s sovereignty, European normative power and enlargement as well as a lasting (geo)political standoff with the ‘West’ following the loss of the Cold War” (p. 17). Similarly, the Russian Federation employs historical-based cleavages and political divisions in foreign countries to favour far-right political parties in those countries, and narratives justifying their domestic and expansionist foreign policy based on fabricated parallelisms with what the Kremlin alleges to be happening in the West.

Russian revisionist narratives refer to specific historical events, periods or refer to unspecified past (e.g., good old times of Slavic unity). Certain narratives concern the whole region (e.g., Soviet ‘liberation’ of CEE from the Third Reich), whilst others can be either country-adjusted (e.g., ‘Polish historical imperialism’) or shared by two or more countries (both Czechs and Slovaks are targeted by revisionist narratives concerning Czechoslovakia).

Table 1 collates examples of such narratives. It is well-worth mentioning that the table aligns itself well with the typology proposed by Bokša that divides Russian narratives in CEE to a few groups concerning: 'Russkiy Mir', Slavic unity, 'Ostalgia', “Anti” rhetoric, and alternative information ( 2019, p. 3) Arguably, in the big picture, all of these baskets can employ historical revisionism and weaponized idealized or manipulated past to conduct information warfare against its targets. The typology does not include the '“Liberation” myth which is one of the cornerstones of the Soviet (and later Russian) propaganda ( Domańska, 2019, p. 4). 

**Table 1.** Examples of Russian revisionist narratives. Source: own study based on the collection of narratives gathered by academia, governments, and think-tanks. The examples of narratives have been selected to represent diverse character of narratives and historical events. ( Arribas *et al.,* 2023a).

Country / Region	Examples of Russian narratives	Historical context	Source initial
Baltic States	*Baltic States were incorporated with the support of local population and in line with international law*	Forceful annexation of Estonia, Latvia and Lithuania into the Soviet Union, 1940	NS1
	*In the years 1940-1949, the Soviet state committed no atrocities against the local [Baltic] populations*	USSR occupation of the Baltics, 1941–1991. Quoted narrative refer to its early period and Stalinist repressions.	NS2
	*“Forest brothers” were murderers, cowards, marauders, criminals, fascists, and collaborators with the* *Nazi regime.*	Soviet fights with the "Forest Brothers", 1950	NS3
	*The trend towards the rehabilitation of Nazism and the heroization of Nazi criminals is increasingly* *taking place in the Baltic States: processions of Waffen-SS legionnaires and their supporters regularly* *take place in Latvia, and annual rallies of SS veterans take place in Estonia (…).*	General / WWII, 1939–1945	NS10
CEE	*The enlargement of NATO and of the European Union were anti-Russian activities*	Enlargement of NATO in 1999 and EU in 2004	NS6
	*During the WWII, the whole CEE region had a "brotherhood in blood" with the Russians*	WWII, 1939–1945	PG1
	*Russia was the primary force that liberated Europe from Nazism*	Fall of the Nazi Germany and Soviet victory, 1945	UM1
Czechia	*Czech Republic is participating in the genocide in Donbas*	Russian aggression on Ukraine and its geopolitical aftermath, 2014	PI1
Czechia and Slovakia	*Nazi-like repression ended the moment Soviet troops entered the Protectorate of Moravia and* *Bohemia*	Fall of the Nazi Germany and Soviet victory, 1945	PI2
	*Perhaps the invasion was a better option than a complete Russophobia into which a Prague Spring* *could have culminated*	Prague Spring, 1968	PC1
	*70% of the Red Army troops who suppressed the Prague Spring were actually Ukrainian*	Prague Spring, 1968	PC2
Estonia	*Joining the Soviet Union was useful for Estonia: in the USSR, higher education was free, and* *representatives of the Union republics had privileges to enrol in leading Russian universities out of* *competition. In the post-war years, Estonia was able to train specialists in Leningrad and Moscow* *higher education institutions.*	Forceful annexation of Estonia into the Soviet Union, 1940	NS9
	*Estonian population has been collaborating with the Nazis to kill Jewish people.*	WWII, 1939–1945	NS8
	*Estonia now holds up these Nazi criminals as local heroes*	Soviet fights with the "Forest Brothers", 1950	NS7
Hungary	*Romanian disrespect towards a Hungarian military cemetery from WWI* [and from WWII - author] *was* *an introduction to a new "Black March" ethnic massacre.*	WWI, 1914–1918 WWII, 1939–1945 The ethnic clashes of Târgu Mureș, 1990	PC9
	*Crimea is Russian, because everybody there* *speaks Russian. This obviously means that* *Szeklerland is Hungarian, because everybody there speaks Hungarian, Upper Hungary of the* *Great Schütt Island is Hungarian, because everybody there speaks Hungarian, similarly to a part of* *Vojvodina, where the majority speaks* *Hungarian*	Treaty of Trianon, 1920, Annexation of Crimea, 2014,	PC18
	*Trianon is not a case closed*	Treaty of Trianon, 1920	PC19
Poland	*II Polish Republic is responsible for its citizens' suffering during WWII*	WWII, 1939–1945	IW1
	*Poland has never abandoned its ‘revanchist’ ambitions towards former Polish territories found in* *Lithuania or the western regions of Ukraine and Belarus*	Polish-Lithuanian Commonwealth, 1569 – 1795 4 ^th^ partition of Poland In 1939	NE1
	*Russia is the only true successor to Kyivan Rus’ because “in the Grand Duchy of Lithuania, other* *processes were unfolding. In the 14th century, Lithuania’s ruling elite converted to Catholicism.* *In the 16th century, it signed the Union of Lublin with the Kingdom of Poland to form the Polish–* *Lithuanian Commonwealth. [..] The process of Polonization and Latinization began, ousting Orthodoxy.*	Polish-Lithuanian Commonwealth, 1569 – 1795	NE5
Slovakia	*Russia being a big Slavic brother, protecting the small Slovak nation from outside (mostly Hungarian)* *oppression*	Historical pan-Slavic unity, mostly XIX	PC20

*1. Trianon Peace Treaty (1920)*

The Treaty of Trianon (4 June 1920) meant the partition of the historical territory of Hungary between the successor states (Czechoslovakia, Romania, Serbia Croatia and Slovenia) with the consequent distribution of the Hungarian population (one third) between the countries of destination. The Trianon topic issue has been a core theme of the Hungarian historiography since then, but its interest has increased in the last decade, being a regular resource in the Hungarian far-right rhetoric ( Braham & Hanebrink, 2020; Kurimay, 2016; Petsinis, 2015; Petö, 2017) and serving a dual purpose: on the one hand, to reinforce nationalism (domestically and between the Magyar minorities abroad) through the exploitation of a victimhood sentiment; and, on the other hand, to contribute to feed anti-Western narratives based on the idea of “historical injustice” committed by the West.

An example of the importance of Trianon memory is found in the legislation adopted on 4 June 2010 by Hungarian Government, named as the ‘Day of National Cohesion’. Cohesion refers to the connections with Hungarian minorities living abroad ( Petö, 2017, p. 7). Petsinis (2015) points out the anti-Western narrative disseminated by Hungarian far-right regarding the “historical injustice” of the Trianon Treaty (1920) and allowed by Western countries, that reduced the Greater Hungary to a third of its former size. Braham & Hanebrink (2020) emphasizes the employment by Fidesz of the emotional legacy of the Treaty as a “collective trauma that unified ethnic Hungarians inside and outside the new borders” (p.3), being well represented in Victor Orban´s memory politics.

The Magyar minority abroad is a hot topic in Fidesz’ foreign policy and can be summed up in the following objectives: providing dual citizenship to Hungarians abroad; assisting the self-preservation of Hungarian communities; supporting minority human rights (such as the preservation of the language or autonomy aspirations) and attending the needs of minorities (cultural, educational, financial…) ( Győri & Syrovátka, 2019). However, although the geopolitical implication of Trianon affected Czechoslovakia, Romania, Kingdom of Serbs, Croats and Slovenes, the bilateral repercussions are no equal, and depend on the political trends and affinities of the current governments towards Hungary. So, minority rights have not a significant effect on relationship with Slovakia or Serbia, both on good terms with Hungary, (Ibid.) while in Romania, with a political sentiment far away Orban government, the open support by Fidesz-KDNP to territorial autonomy for the Szeklers (ethnic minority) (székely) supposes a latent bilateral issue.

*2. Second World War*

The Second War World is a well-documented issue within Russian propaganda ( Belavusau *et al.,* 2021; Khaldarova & Pantti, 2016; Sherlock, 2016; Vázquez Liñan, 2010). The Victory Day, as a symbol of the defeat of the Nazi Regime at hands of the Soviet Union acted as pivotal for Soviet identity construction, conceptualising the Soviet Union as a guarantor of freedom and sovereignty ( Luxmoore, 2019) and for supporting Soviet nationalism and patriotism ( Vujacic, 2007).

Today this rhetoric is maintained by the Kremlin with the purpose of creating a favourable common memory across all the post-Soviet space and serving to reinforce patriotism, being a key theme in the current educational curricula ( Kratochvíl & Shakhanova, 2020). The memory of World War II is also strongly connected with Vladimir Putin´s cult of personality ( Vázquez Liñan, 2010).

The importance of World War II became evident in the 2020 Constitutional Referendum that elevated “this pillar to the realm of mnemonic constitutionalism” ( Belavusau *et al.,* 2021, p. 15). The novel Article 67.1 of the Russian constitution consecrated the protection of “historical truth and the respect of the ‘memory of the defenders of the Fatherland’ targeted mainly the Soviet past and its commemoration, in particular the glorification of the Soviet army” ( Belavusau *et al.,* 2021, p. 15).

References to World War II in official statements are also habitual in contemporary Russia ( Kratochvíl & Shakhanova, 2020). An illustrative example is a Putin´s article published in the US conservative magazine ‘The National Interest’, and in advance of the Russian Constitutional Russian. The article entitled ‘The Real Lessons of the 75th Anniversary of World War II’ ( Putin, 2020) contains the main points developed by the Kremlin regarding memory construction of the World War II; that is to say: the defeat of the Nazi Regime was the result of the collective effort of all the people that belonged the Soviet Union; the Munich Agreement (1938) was the real trigger of the War; dilutes the Russian responsibilities associated to the Molotov-Ribbentrop Pact (an agreement signed with Nazi Germany in August 1939) accusing simultaneously the West, especially the United Kingdom and the United States for its contribution to the war through financial and industrial enterprises that invested in the German defence industry (Ibid.).

In a speech delivered in December 2019, addressing the leaders of remaining CIS countries, Putin argued that it was not the 1939 German-Russian Pact, but the 1938 Munich agreement, the so-called by himself “Munich betrayal” what made possible the Second World War ( Sherlock, 2016). The condemnation of the Molotov-Ribbentrop pact by the Supreme Council of the Union of Soviet Socialist Republics (USSR) in a decree of September 23, 1989 ( Juurvee, 2021), is maintained by Putin, but he has introduced some nuances regarding the secret protocols that are described as “an act of personal power,” which in no way reflects “the will of the Soviet people” ( Putin, 2020).

Another fundamental rhetorical resource for the Kremlin, related to the so-called Great Patriotic War, is the well-known narrative of the fascist threat. The evocation of a heroic image towards the Soviet Red Army supported by the Russian population against the fascist enemy, is a resource employed since Stalin to constraint domestic uprisings ( Luxmoore, 2019).

The Victory Day, celebrated each year on 9 ^th^ May, acts in a symbolic level as a reminder of the victory over fascism and of the price the USSR paid. In the 2000´s, the same elements can also be identified in the “preventive counterrevolution” strategy designed by Gleb Pavlosky, former Kremlin´s ideologue and advisor, as a response to Ukraine´s Orange Revolution (2004) (Ibid.) This strategy is behind the creation of Nashi movement, the Anti-Orange Committee and Antimaidan and included together actors of various political sensibilities –Soviet nostalgists such as Kurginyan, Eurasianists such as Dugin, Stalinists like Prokhanov– all “united in the view that an opposition aided by the West represents a threat to Russia’s sovereignty” ( Luxmoore, 2019, p. 833).

On the other hand, recent memory laws within some CEE countries positioned WWII as a core theme, bringing to the surface tensions between neighbours that are rooted in the past. Belavusau *et al.* (2021) conducted a comparative analysis on the historical memory legislation enacted in Poland, Ukraine and Russia in the context of memory wars and the historical narratives that are employed as political contestation among the three countries and the potential conflicts derived from them.

In the case of Poland and Russia, unsolved historical conflicts include the Soviet aggression against Poland in 1939, the Katyń massacre of nearly 22,000 Polish officers in 1940, and the four decades of communism installed by the USSR. The Ukrainian memory lies on the trauma of the Soviet repression during Stalinism. In recent years, these disputes over history have been reconfigured into an open armed conflict since the annexation of Crimea and Sevastopol and the more recent invasion of Ukraine ( Belavusau *et al.,* 2021, p. 10).

*3. The memory of Communism*

Russian manipulated history develops the false idea that the Baltic States were voluntarily incorporated into the USSR in 1940. The soviet period is also shown positively and remarking the excellence of higher education ( Sazonov, 2021). 

On May 2020, the Presidents of the Baltic States signed a joint statement in the context of the 75th anniversary of the end of World War II in Europe. Estonia, Latvia and Lithuania made clear the coercive nature of their incorporation into the USSR.

“The central and eastern part of the continent remained under the rule of communist regimes for almost half a century. The Soviet Union used overwhelming military force, indiscriminate repression, mass deportations and total ideological control to subjugate the Baltic nations.
  The Soviet occupation continued until the collapse of the Soviet Union, thanks to the peaceful and determined efforts of our citizens in our territories and throughout the world, national independence of Estonia, Latvia and Lithuania was restored in 1990–1991. A few years later, the Russian army was withdrawn from our states.” ( Karis, 2020)

In Estonia, the Internal Security Service (KAPO) referred to “History in Russia’s influence operations” ( Estonian Internal Security Service, 2014, p. 10; Estonian Internal Security Service, 2018, p. 14). With regard to Latvia, Engizers (2021) considers Russia’s abuse of history through “accusations of the falsification of history” as the most significant aspect of its intervention ( Engizers, 2021, p. 43). This author notes that, “from adoption of the first constitutional acts in early 1990, Latvia developed its statehood grounded on the idea of continuity of state and the understanding that being within the USSR had been the result of illegal occupation” ( Engizers, 2021, p. 44). For his part, Russia’s National Security Strategy considers the national security implications of those attempts to revise Russia’s history ( Engizers, 2021, p. 45).

***Agency of Russian and pro-Kremlin amplifiers.*** Russian political history is centralised and developed in Kremlin but is spread throughout a distributed network of diverse institutions ( Domańska & Rogoża, 2021, p- 40). Certain disinformation-observers underline that it predominantly uses official news agencies, alternative platforms and local political actors – instances of which have been observed in Czechia, Hungary and Slovakia ( Győri & Syrovátka, 2019). In the more granular level, this toolbox consists of:

a) Russian Ministry for Foreign Affairs, through its embassies and diplomatic missions. To show an example of their employment, in 2019 Russian embassies launched an Internet campaign under the hashtag #truthaboutwwii where it promoted a ‘Russian interpretation’ of history of the World War II. It has been disseminated, in particular by Russian diplomatic offices in the USA, Canada, the Republic of South Africa (RSA), the Organization for Security and Co-operation in Europe (OSCE), Houston mission to the United Nations (UN), Israel, Estonia, and Australia ( Schafer, 2019)
b) Russian national and international media – especially Russia Today (RT) and Sputnik ( Domańska & Rogoża, 2021)
c) Conspiracy and fringe websites that spread misinformation and pro-Kremlin views and are, arguably, the most dangerous ( Vejvodová, 2017)
d) Russian government organized non-governmental organizations (GONGOs) and higher education entities (such as the Russkiy Mir foundation), which operate abroad. For instance, the Russian Cultural and Educational Centre opened in Pecs and in Debrecen, Hungary, in April 2017 ( Bartha, 2017)
e) Russian Orthodox Church ( Bartha, 2017)
f) Civil society organisations that amplify Russian narratives but otherwise claim no organisational no financial link to Russia (non-governmental organizations (NGOs) and GONGOs ( Domańska & Rogoża, 2021)
g) Societies of ‘patriotic’ historians ( Domańska & Rogoża, 2021)
h) Kremlin-funded political parties ( Domańska & Rogoża, 2021)
i) Agents of influence, and political trolls and bots
j) Popular culture

Their operations are mainly funded by the Russian state treasury, state enterprises, and Russian private companies ( Domańska & Rogoża, 2021). Additionally, it is financed by other means, such as website traffic revenue (e.g., owing to number of times it was seen on social media), targeted political ads ( Havlíček & Yeliseyeu, 2021, p. 96), other states’ funding (such as the 7.7 million EUR support for the Russian Orthodox Church secured by Hungary) ( Bartha, 2020) or even private actors from abroad (as in the case of the Hungarian diaspora media) ( Győri & Molnár, 2020).

Russia disseminates its narratives across the aforementioned channels in a recurring (or even circular) ( Domańska & Rogoża, 2021, p. 38) way as it constitutes a fixed part of the information warfare ( Karpchuk, 2021; U.S. Department of State factsheets, 2022). In this, it exists independently of the actual history, even if specific narratives use contemporary events as a pretext and good momentum to re-surface. Examples of the latter include historical anniversaries, such as 75. Anniversary of liberation of Auschwitz-Birkenau in 2020, and related commemoration of Victory Day in Moscow in the same year ( Baluk & Demczuk, 2020, p. 17–18). These occasions have brought a visible engagement of Russian political leaders who underlined the role of Russia in liberation from fascism, and to re-iterate its unique role as a primary victory over fascism, and speculations of the Euro-Atlantic tacit agreements with the Third Reich (symmetric to Ribbentrop-Molotov pact) voiced in the article written by the President Putin in the National Interest in June 2020 ( Domańska & Rogoża, 2021, p. 37).

***Intent and political context.*** As summed-up by Medveded (2021), the intention behind the Russian historical revisionism is to “construct a consolidated history narrative stressing greatness and military victories and whitewashing the crimes of the past, like Stalin’s atrocities (e. g. the Katyn’ massacre of Polish officers in 1940) and Soviet colonial aggression (Prague Spring 1968, war in Afghanistan in 1979–1989).” ( Medveded, 2021, p. 25)

The aspiration of the Kremlin to be seen as the main victor over fascism translates into its alleged right to be considered as a global power free to have a say in its ‘near’ and ‘far’ abroad. It also demands from its Euro-Atlantic partners to dismantle international blocs into a single international order, which would also diminish the role of NATO ( Domańska, 2015).

However, its instrumentalised interpretation of history – both generalized or relating to actual events – in many cases stays in sharp contrast with national and collective memories of CEE countries. For instance, the USSR engagement in suppressing the Hungarian uprising of 1956 claimed that it was essentially an anti-fascist intervention ( Poellath, 2023, p. 15). Denial of Russian historical revisionism attempts meet with hostile responses from the Russian government, such as its attack on the European Parliament’s resolution of 2019 ( Domańska & Rogoża, 2021, p. 35) or launching the #truthaboutwwii campaign online.

***Estimated effects and CEE resilience.*** Throughout the past two hundred years, any of the Viségrad and Baltic States has either been politically subordinated or directly incorporated to Russia. Moscow has drawn them into its orbit in the aftermath of the partition of the Polish-Lithuanian Commonwealth in XVIII century or after World War II, when it consolidated its dominance in Central and Eastern Europe with the creation of the socialist block. This has further perpetuated the image of Russia as an oppressive and hostile country, especially in the Baltics and in Poland ( Milo, 2021, p. 9). For instance, Polish society remains suspicious of pro-Russian propaganda, and its attempts to go mainstream enjoyed only a limited success ( Havlíček & Yeliseyeu, 2021, p. 177). Moreover, Poland - together with Estonia, Latvia, and Lithuania - has been qualified by Petar Kurečić (2017, p. 64–65) as a New Cold Warrior, what translates into vigorously anti-Russian sentiments both on diplomatic and public opinion levels. 

Other countries of the region demonstrate a much more benign attitude towards Russia, falling into the categories of “bear-huggers” (Slovakia) or “bear-feeders” (Hungary and Czechia) ( Milo, 2021, p. 9). GLOBSEC’s conclusions find themselves in line with Kurečić’s typology where Hungary, Slovakia, and Czechia (albeit the latter with a question mark) were qualified as Pragmatics ( Kurečić, 2017, p. 64–65), demonstrating a lesser degree of negative sentiment towards Russia. Despite these differences, the study ( Hlatky, 2021, p. 21) shows that even in Slovakia the popular consumption of disinformation is low and averages 14.5% of readers. The study paints an optimistic, yet nuanced picture, and underlines that disinformation outlets are also less trusted than mainstream news sources.

The Russian historical revisionism in CEE attempts to sell an image of Russia as a protector and liberator, while either whitewashing its crimes or attacking its regional competitors. Its main intention is to safeguard the Russian civilization exceptionalism and to secure its unquestionable position on the international area – delegitimizing Euro-Atlantic influences in what it considers its “Near Abroad”. To achieve that it uses a complex distributed network consisting of various actors and channels, that are predominantly financed by Russia – either directly or through proxies. Its outcome shows a rather limited impact with few successes, plausibly due to its exposure to a distinct and living collective memory.

### Analysis of the EUvsDisinfo Database

The European public opinion witness during the last years an unprecedented torrent of Russian historical revisionism (See for instance: EUvsDisinfo’s Lukashenka’s Belarus celebrates the Soviet attack on Poland).

Historical revisionism is the method used in the context of Russian and pro-Kremlin disinformation and influence efforts. This is the case of the European History and the Trans-Atlantic relation too. Recurring accusations from pro-Kremlin media provide different narratives for various audiences. Some of these narratives have been in use for decades, such as the presumed Nazi behaviour of different states or the role of Russia in World War II. The same instruments are popular in Russia: new-old historic events are given a new face and the masses are encouraged to rally behind the flag for new reasons. Narratives are combined and modified based on current events and prevailing attitudes, in order to legitimize the Kremlin’s actions and narratives.

By querying the EUvsDisinfo database through search terms such as “historical revisionism” a number of 408 items were discovered for the period 2016–2022, with 10 January 2022 as the date of the latest disinformation item collected: 244 items resulted from the terms “historical revisionism WWII”; of which, 149 for the term “historical revisionism WWII Poland”; 71 results for “historical revisionism West”; 41 results for “historical revisionism EU”, and 16 results for “historical revisionism NATO”. Through the search term “Yugoslavia” we were able to discover 16 relevant items.

We identified seven principal themes:

*1. West aggressive intentions against Russia, including the EU (with the focus on France and Germany relation), and EU-NATO relations.*

The West aggressive intentions, the European responsibility for some events in USSR and EU and NATO actions linked to Nazi behaviour are only some of the distorted messages spread by the pro-Kremlin media. The Kremlin’s policy of historical revisionism accuses the West and particularly EU members, such as the Baltic States and Poland of the “falsification and re-writing” of World War II history. European states are continuously accused of their support for the Nazi or Fascist ideology. Any disagreement with the Kremlin's official view on the history of World War II is automatically labelled by Russia as support for “Nazism” or “falsification of history”; including claims such as “the West tries to revise history, questioning the decisions of the Nuremberg trial” (Article 1, Table 2).

**Table 2.** EUvsDisinfo database´s articles referenced in the text.

Article 1. EUvsDisinfo. *The West tries to revise history, questioning the decisions of the Nuremberg trial*. https://euvsdisinfo.eu/card/?url=the-west-tries-to-revise-the-history-questioning- the-decisions-of-the-nurnberg-tribunal
Article 2. EUvsDisinfo. *The West has aggressive intentions against Russia and its allies just as in 1938*. https://euvsdisinfo.eu/report/the-west-has-aggressive-intentions-against-russia-and- its-allies-just-as-in-1938
Article 3. EUvsDisinfo. *The West uses Ukraine to destroy Russia like it used the nazi Germany to destroy USSR.* https://euvsdisinfo.eu/report/the-west-uses-ukraine-to-destroy-russia-like- it-used-the-nazi-germany-to-destroy-ussr
Article 4. EUvsDisinfo. *Russia has been dividing Ukraine with NATO for the past 300-400 years.* https://euvsdisinfo.eu/report/russia-has-been-dividing-ukraine-with-nato-for-the-past
Article 5. EUvsDisinfo. *The army of the EU attacked the Soviet Union and then was defeated in 1945. https://euvsdisinfo.eu/report/the-army-of-the-eu-attacked-the-soviet-union-and*
Article 6. EUvsDisinfo. *The Baltic States are the vanguard of the anti-Russian movement in the West*. https://euvsdisinfo.eu/report/the-baltic-states-are-the-anti-russian-vanguard-of-the- west
Article 7. EUvsDisinfo. *For the West, Russia is an enemy that must be surrounded with military bases.* https://euvsdisinfo.eu/report/for-the-west-russia-is-an-enemy-that-must-be- surrounded-with-military-bases/
Article 8. EUvsDisinfo. *The USA and NATO carry out a propaganda war around WWII history in order to defame and isolate Russia and President Putin*. https://euvsdisinfo.eu/report/the-usa- and-nato-carry-out-a-propaganda-war-around-wwii-in-order-to-defame-and-isolate-russia-and-president-putin
Article 9. EUvsDisinfo. *Western revisionism targets Putin.* https://euvsdisinfo.eu/report/western-revisionism-targets-putin
Article 10. EUvsDisinfo. *The West keeps following Joseph Goebbels's tips to destroy history*. https://euvsdisinfo.eu/report/the-west-following-joseph-goebbelss-tips-to-destroy-history/
Article 11. EUvsDisinfo. *The present ruling elites of the West follow many concepts of Nazi Germany.* https://euvsdisinfo.eu/card/?url=german-and-french-military-cooperation-creates- contours-for-the-4th-reich
Article 12. EUvsDisinfo. *The West is a terrorist structure*. https://euvsdisinfo.eu/report/the-west-is-a-terrorist-organization-like-al-qaida-or-daesh-and-it-is-terrorizing-the-russians-as- much-as-the-nazis-terrorized-the-jews-during-wwii/
Article 13. EU vs. Disinfo. *Russophobic fascist west wants Ukraine to destroy Moscow.* https://euvsdisinfo.eu/report/rusophobic-fascist-west-push-ukraine-to-conduct-a-war-against-russia/
Article 14. EUvsDisinfo. *The US and EU countries are Russophobic, fascist and nazi regimes* https://euvsdisinfo.eu/report/the-us-and-eu-countries-are-russophobic-fascist-and-nazi- regimes/
Article 15. EUvsDisinfo. *Historically, the West has always been barbaric and similar to ISIS “the West has never progressed beyond barbarity and human prehistoric times which included the* *periods of slavery, feudalism and capitalism”* https://euvsdisinfo.eu/report/historically-the-west-was-always-barbarian-and-similar-to-isis/
Article 16. EUvsDisinfo. *Brussels uses Russophobia as a uniting idea to prevent the EU's collapse*. https://euvsdisinfo.eu/report/brussels-uses-russophobia-as-a-uniting-idea-to-prevent- the-eus-collapse/
Article 17. EUvsDisinfo. *European Union is spreading anti-soviet myths about the beginning of World War II “to distort the historical truth about the outbreak of World War II European Union* *and spread anti-Soviet myths about the beginning of World War”* https://euvsdisinfo.eu/report/european-union-is-spreading-anti-soviet-myths-about-the-beginning-of-world-war-ii
Article 18. EUvsDisinfo. *EU's historical revisionism contributes to Neo-nazi trends in Ukraine and the Baltic States.* https://euvsdisinfo.eu/report/eus-historial-revisionism-contributes-to-neo- nazi-trends-in-ukraine-and-the-baltic-states
Article 19. EUvsDisinfo. *The Baltic States are perpetuating the myth of the Soviet occupation.* *https://euvsdisinfo.eu/report/baltic-states-are-perpetuating-the-myth-of-the-soviet-occupation/*
Article 19. EUvsDisinfo. *The EU parliament and the OSCE promote false history.* https://euvsdisinfo.eu/report/the-eu-parliament-and-the-osce-promote-false-history
Article 20. EUvsDisinfo. *Charles de Gaulle defended the project of Europe from the Atlantic to the Pacific oceans.* https://euvsdisinfo.eu/report/charles-de-gaulle-defended-the-project-of- europe-from-the-atlantic-to-the-pacific-oceans
Article 21. EUvsDisinfo. Creation of NATO was not justified. https://euvsdisinfo.eu/report/creation-of-nato-was-not-justified
Article 22. EUvsDisinfo. *If it was not for Stalin, France wouldn´t be one of the great nations.* https://euvsdisinfo.eu/report/if-it-werent-for-stalin-france-wouldnt-be-one-of-the-great-nations/
Article 23. EUvsDisinfo. *Americans rewrite and forget their history because of protests against racism.* https://euvsdisinfo.eu/report/americans-rewrite-and-forget-their-history-because-of- protests-against-racism
Article 24. EUvsDisinfo. NATO lies about the reasons for the fall of the Berlin Wall. https://euvsdisinfo.eu/report/nato-lies-about-the-reasons-for-the-fall-of-the-berlin-wall
Article 25. EUvsDisinfo. *The occupation regime, introduced by the victorious countries in Germany.* https://euvsdisinfo.eu/report/the-occupation-regime-introduced-by-the-victorious- countries-in-ge
Article 26. EUvsDisinfo. *Ukrainian nationalists want to get rid of everything connected to Russia.* https://euvsdisinfo.eu/report/ukrainian-nationalists-get-rid-of-everything-connected-with- russia
Article 27. EUvsDisinfo. *The Molotov-Ribbentrop Pact played no role in unleashing World War II.* https://euvsdisinfo.eu/report/the-molotov-ribbentrop-pact-played-no-role-in-unleashing- world-war-ii/
Article 28. EUvsDisinfo. *USSR is not to blame for the beginning of World War II.* https://euvsdisinfo.eu/report/ussr-is-not-to-blame-at-the-beginning-of-world-war-ii/
Article 29. EUvsDisinfo. *USSR tried to prevent the start of World War II, but Europe abandoned the anti-Hitler coalition*. https://euvsdisinfo.eu/report/the-ussr-tried-to-prevent-the-start-of- wwii-but-europe-abandoned-the-anti-hitler-coalition
Article 30. *EUvsDisinfo. In 1938, Poland was a nazi ally.* https://euvsdisinfo.eu/report/in-1938-poland-was-a-nazi-ally/
Article 31. EUvsDisinfo. *Poland reached mutual agreement with nazi Germany and participated in the partition of Czechoslovakia after the Munich pact.* https://euvsdisinfo.eu/report/ poland-reached-a-mutual-agreement-with-nazi-germany-and-participated-in-the-partition-of-czechoslovacchia-after-the-munich-pact
Article 32. EUvsDisinfo. *Poland was not an innocent victim of Nazi aggression.* https://euvsdisinfo.eu/report/poland-was-not-an-innocent-victim-of-nazi-aggression
Article 33. EUvsDisinfo. *The Molotov-Ribbentrop pact is being demonized by the european countries.* https://euvsdisinfo.eu/report/the-molotov-ribbentrop-pact-is-being-demonized-by- the-european-countries
Article 34. EUvsDisinfo. *Poland and Nazi Germany planned a military campaign against the Soviet Union and the final solution.* https://euvsdisinfo.eu/report/poland-and-nazi-germany- jointly-planned-a-military-campaign-against-the-soviet-union-as-well-as-the-final-solution-to-the-jewish-question
Article 35. EUvsDisinfo. *Nazi-Soviet pact was not the cause of WWII*. https://euvsdisinfo.eu/report/nazi-soviet-pact-was-not-the-cause-of-wwii
Article 36. EUvsDisinfo. *The USSR signed the Ribbentrop-Molotov pact with nazi Germany due to Poland´s aggresive foreign policy.* https://euvsdisinfo.eu/report/ussr-signed-ribbentrop- molotov-pact-nazi-germany-poland-aggressive-foreign-policy/#:~:text=Historical%20documents%20newly%20released%20by%20Russia%E2%80%99s%20Ministry%20of,pose%20a%2 0really%20serious%20threat%20to%20the%20USSR
Article 37. EUvsDisinfo. *Poland’s refusal to allow Soviet troops to enter their territory led to Molotov-Ribbentrop pact*. https://euvsdisinfo.eu/report/poland-was-not-an-innocent-victim-of- nazi-aggression
Article 38. EUvsDisinfo. *Red Army entering Poland did not plan to seize this country.* https://euvsdisinfo.eu/report/red-army-entering-poland-did-not-plan-to-seize-this-country
Article 39. EUvsDisinfo. *US presence in Europe, portraying Europe as a vassal of the USA and trying to undermine the sovereignty of European countries.* https://euvsdisinfo.eu/report/the- leaders-of-european-countries-are-subject-to-american-dictates
Article 40. EUvsDisinfo. *Europe is a military colony of the United States and European countries are obedient vassals of the Americans*. https://euvsdisinfo.eu/report/europe-is-a-military- colony-of-the-united-states-and-european-countries-are-obedient-vassals-of-the-americans
Article 41. EUvsDisinfo. *About two thousand civilians were killed in NATO´s bombing of Yugoslavia*. https://euvsdisinfo.eu/report/about-two-thousand-civilians-were-killed-in-natos- bombing-of-yugoslavia
Article 42. EUvsDisinfo. *Poland wanted to ally with Germany and attack the USSR.* https://euvsdisinfo.eu/report/poland-wanted-to-ally-with
Article 43. EUvsDisinfo. *NATO lies about the reasons for the fall of the Berlin wall*. https://euvsdisinfo.eu/report/nato-lies-about-the-reasons-for-the-fall-of-the-berlin-wall

The recurring pro-Kremlin narrative on Western belligerence (the West as a whole) towards Russia and its allies, such as Belarus or the supposedly hostile anti-Russian intentions and policies of the aggressive West, are presented through messages such as:

“The West has aggressive intentions against Russia and its allies just as in 1938” (Article 2, Table 2); “the West wants to destroy Russia, just as it did in 1938 through the Munich Agreement … Now it uses Ukraine” (Article 3, Table 2); “the West also does this through sanctions, political pressure and NATO exercises” (Article 3, Table 2); “Russia has been dividing Ukraine with NATO for the past 300–400 years” (Article 4, Table 2); “The army of the EU attacked the Soviet Union and then was defeated in 1945” (Article 5, Table 2); “The Baltic States are the vanguard of the anti-Russian movement in the West” (Article 6, Table 2); “for the West, Russia is an enemy that must be surrounded with military bases” (Article 7, Table 2); “the USA and NATO carry out a propaganda war around WWII history in order to defame and isolate Russia and President Putin” (Article 8, Table 2); “Western revisionism targets Putin” (Article 9, Table 2), etc.

Some of the messages are aimed at discrediting the EU as a whole, creating an illogical connection between Hitler’s Nazi Germany and the European Union. Thus, the accusation of Nazism/ Fascism and linking the EU to Nazi Germany is a recurring technique of pro-Kremlin disinformation outlets aimed at discrediting the West:

“The West keeps following Joseph Goebbels's tips to destroy history” (Article 10, Table 2); “The present ruling elites of the West follow many concepts of Nazi Germany” (Article 11, Table 2); “the West is a terrorist structure” (Article 12, Table 2), “the West is fascist” (Article 13, Table 2), and that “the US and EU countries support Russophobic, neo-fascist, terrorist political parties and movements in the post-Soviet space” (Article 14, Table 2), “the West has never progressed beyond barbarity and human prehistoric times which included the periods of slavery, feudalism and capitalism” (Article 15, Table 2); “Brussels uses Russophobia as a uniting idea to prevent the EU's collapse” (Article 16, Table 2); etc.

Russia had repeatedly criticized other countries for their attempts to rewrite history. In German, Czech, Greek, Polish or French, pro-Kremlin media outlets such as de.rt.com, RT Online – Facebook, sputniknews.gr spread the distorted info about the West deliberate campaign of revising World War II history in order to change the perception of the USSR and also of Russia. Thus, in December 2019, the European Parliament tried “to distort the historical truth about the outbreak of World War II European Union and spread anti-Soviet myths about the beginning of World War” (Article 17); or in November 2021 the EU was accused to contribute “to neo-Nazi trends in Ukraine and the Baltic States” (Article 18, Table 2).

Moreover, on June 2020, the Kremlin’s policy of historical revisionism, accuses the US, EU and NATO of the “falsification and re-writing” of World War II history in order to harm Russia (Article 19, Table 2). The same article makes another claim about the European Parliament and the OSCE promoting false history.

The recurring pro-Kremlin narrative according to which the West is trying to rewrite World War II history, is often fuelled by another narrative, accusing the West of Russophobia (anti-Russian sentiment). The narrative is part of the Kremlin’s own practice of historical revisionism, in which all views and interpretations of past events not in accordance with an official state history are to be dismissed, censured, and discredited.

There are only some disinformation cases about the EU-NATO relation. In January 2022, in the new international context, fr.sputniknews.com spread the news that “Charles de Gaulle defended the project of Europe from the Atlantic to the Pacific oceans” (Article 20, Table 2). According to EUvsDisinfo, this is a historical manipulation case which aims to make the Eurasian political vision of the Kremlin acceptable by Europeans. The quote is not from Charles de Gaulle but from Vladimir Putin. “Charles de Gaulle referred several times in his speeches to Europe from the Atlantic to the Ural, a geographical reference rather than a political one at a time when the iron curtain separated Europe from two opposite sides.” (Article 20, Table 2) The Eurasian political vision of the Kremlin is presented through historical manipulation, so that it can become acceptable by Europeans.

In the same idea, on November 2019, the Russian outlet vesti7.ru states that “Macron thinks that NATO was conceived as a response to the enemy, as a response to the Warsaw Pact” (Article 21, Table 2) but in 1949 the Warsaw Pact did not exist, thus the NATO had no justification, according to Russian propaganda. On other disinformation cases, the USSR is presented as the main supporter of France during World War II, when the US and England supposedly did not anticipate a victory for France. France’s victory is, as such, presented as “a direct result of Stalin’s behest, through both the USSR’s and personal involvement” (Article 22, Table 2). Furthermore, France’s position as one of the five founding countries of the UN is also presented as a result of Stalin’s and USSR’s support.

Few cases are about the Western values: “Americans rewrite and forget their history because of protests against racism” (Article 23, Table 2); “NATO lies about the reasons for the fall of the Berlin Wall” (Article 24, Table 2). Moreover, Petr Tolstoy, Channel One´s host show “Vremya Pokazhet”, promotes on his YouTube channel (This file has been removed from Youtube), a still-present occupation regime in Germany, as a result of the victory in World War II, placing the state as defeated and without sovereignty (Article 25, Table 2).

Websites available in targeted countries are used, such as fr.sputniknews.com, pl.sputniknews.com, pl.rubaltic.ru, vesti7.ru, vesti.ru, news-front.info, or Geopolitica.ru. Also, social media is a present channel for disseminating the messages, such as YouTube - Rossia 24, ar.rt.com, and also television channels, such as ren.tv. The media channel that seems to be prominent is sputniknews.com. Moreover, this channel appears also when the messages are delivered in other languages such as French, Russian, Arabic, Polish, and German. The information manipulation techniques employed are the distortion of the historical truth; the accusations that some countries (Ukraine, Poland, and Baltic States) associate themselves with Hitler; the framing of Russia and its actions positively; framing Russia as a victim of the West.

*2. Moscow reclaiming its ‘“zone of influence’” by denying the Soviet occupation in the neighbourhood or accusing the former Soviet states, especially the Baltic States (Lithuania, Latvia and Estonia), of historical revisionism, Russophobia, and violation of human rights.*

Russia’s messages are based on distorted historical realities, claiming, for example, that Crimea became part of Ukraine by accident, at a time when the Ukrainian statehood was a distant possibility. On this note, Russia uses the denial of the historical facts, claiming, for example, that Ukraine has never existed as an independent country or that the Belarusian nation was artificially created by the Soviet Union. Another used strategy is to highlight the Soviet contribution to the development of the neighbouring countries, presenting itself as a peacemaker and liberator.

Russia hopes that the propaganda narratives will attract more support from both Russian nationals and people from countries that were previously under the Russian influence. However, it seems that Russia manages to achieve the opposite effect as it becomes more isolated from the West and, implicitly, from its former sphere of influence. The main targeted audience are the Russian people and the citizens of the countries that were under Moscow’s sphere of influence.

Russia’s intentions are to (re)constitute its former sphere of influence but very few countries seem to willingly accept Russia’s domination as most of the time, Russia’s security was a two-sided coin, meaning insecurity to the countries that accepted Moscow’s influence. Moreover, Russia considers that the bordering countries should be grateful to Russia as USSR protected them from external challenges and threats. The intention is to gather social support for present and future actions aimed at (re)gaining more influence in the post-Soviet sphere. The messages are part of the Kremlin’s policy of historical revisionism and imperialism. Some of the messages are delivered in the context of different celebrations, such as the commemoration of the defeat of Nazi Germany on May 8 (Victory in Europe (V-E) Day) or the anniversary of World War II. Stalin’s rehabilitation techniques also result from the information presented, such as building 20,000 churches in Georgia after World War II, or as defeating Nazism.

*3. Denying Ukraine Nation and Statehood and using the historical revisionism to justify Crimea, the Eastern occupation, and the war.*

The Russian state sponsored media outlets claim that Ukraine was created artificially and, moreover, that Russia and Ukraine are a single country that was turned into two parts by the enemies. In 2016, 2017, and 2018, the narratives were focused on the independence of Ukraine. In some cases, the messages mentioned that Ukrainian towns are waiting for an external power to protect them from the Ukrainian government. In 2019, the messages disseminated by Russia questioned the history of the Ukrainian statehood. At the same time, the disinformation narrative promoted the idea that Ukraine started disrupting all economic ties with Russia and opened its markets for EU products. In 2020, Kremlin’s messages were focused on the fact that Ukraine wants to rewrite its history and to move away from Russia. The illegal annexation of Crimea was a recurring narrative, with Russia accusing Ukraine of spreading distorted information on Donbass and Crimea. In 2021 and 2022, at the core of Russia’s disinformation messages stood the fact that Ukraine is not a real state and moreover, it was created by the Bolsheviks.

The aim of the disinformation narratives promoted by Russia is to undermine the statehood of Ukraine and misrepresent the relations with the West. For this purpose, Ukraine is presented as a historical part of Russia, a country without history or state institutions. Among the key messages transmitted, we can identify the denial of the Ukrainian nation and statehood and the claim that the Ukrainian state was created by the Bolsheviks. In another key message, Kremlin claims that Ukraine is under external control and that the West are attempting to tear Ukraine apart from Russia and to make it anti-Russian.

The disinformation narratives include specific historical events. One of the examples is the news about the demolition of the Soviet Marshal Georgiy Zhukov monument in Ukraine. 

In March 2019, Rossia 24 Youtube Chanel announced the destruction of this monument “by nationalists and radicals whose purpose is to get rid of everything connected to Russia” (Article 26, Table 2). The EuvsDisinfo disproof explain that the monument “falls under the action of the law on decommunization, which recognizes the communist totalitarian regime of 1917–1991 in Ukraine as criminal and pursuing a policy of state terror” (Article 26, Table 2), the law requiring the dismantling of monuments or objects that commemorate that period.

The news is mostly delivered in the Russian language through Russian media channels such as Rossiya 24, Russia Today, Sputnik, Tvzvezda, Tsargrad, or through YouTube channels such as Voskresnyi vecher s Vladimirom Solovyovym, and Pervyy kanal. The dissemination of disinformation narratives is mostly in the Russian language, the prominent media channel for delivering the messages being Rossia 24 and Voskresnyi vecher s Vladimirom Solovyovym – YouTube.

The information manipulation techniques employed are the distortion of the historical truth regarding the statehood and independence of Ukraine; blaming Ukraine for wanting to destroy its historical connections to Russia; accusing Ukraine of spreading false information about Crimea and Donbass. The disinformation messages are mostly delivered in the Russian language, which shows that they are prominently directed towards Russian citizens rather than towards the people living in neighbouring countries. This does not come as a surprise, as Russia is trying to convince its population that Ukraine is, in fact, part of the country.

*4. Disinformation cases about WWII and the Molotov-Ribbentrop Pact.*

The Russian disinformation campaign about the WWII promote the official Russian historiography which is considered the only ‘true’ way of interpreting the historical events about the war ( Teodor & Teodor, 2021, 173). To support any interpretation or affirmation the events which lead up to the Second World War, no documentary evidence and no dates are given. The technics used are: “promoting false narratives about the European past, misinterpreting, twisting, or omitting important key facts in order to present victims as oppressors and oppressors as victims” ( Teodor & Teodor, 2021, 173). The intention is to reinforce common pro-Kremlin disinformation narratives about World War II and the Molotov-Ribbentrop pact like: “The Molotov-Ribbentrop Pact played no role in unleashing World War II” (Article 27, Table 2”) and “USSR is not to blame for the beginning of World War II” (Article 28); “The USSR tried to prevent the start of World War II, but Europe abandoned the anti-Hitler coalition” (Article 29, Table 2); “Poland should blame itself for the German-Soviet attack in September 1939” (Article 30, Table 2). EUvsDisinfo experts disproof and promote the historical facts about the Molotov-Ribbentrop Pact and the beginning of the WWII.

*5. The underestimation of URSS responsibility over the political developments in the Polish People’s Republic and Poland seen as rewriting the history*

The Pro-Kremlin media routinely use historical revisionism to denigrate and attack Poland, which became from March 2019 the central of the narratives about World War II and Molotov-Ribbentrop Pact. Thus, Poland is accused of the “falsification and rewriting of its history” for political goals (especially the history of World War II) and, sometimes, is presented as a supporter of Nazi ideology (Article 31, Table 2, Article 32, Table 2).

EUvsDisinfo highlights the principal Russian narrative about Poland: “Poland was not an innocent victim of Nazi aggression” (Article 32, Table 2); “Poland was also an ally of Germany. Moscow had no other choice than signing the Molotov-Ribbentrop Pact (Article 33, Table 2); “Poland and Nazi Germany planned a military campaign against the Soviet Union and the final solution” (Article 34, Table 2); “The responsibility for WWII lies with the Polish elite” (Article 35, Table 2).

Some fake historical documents released by Russia’s Ministry of Foreign Affairs are promoted as evidence for the lack of aggressivity in the Soviet foreign policy at the beginning of the WWII when decided to sign the Ribbentrop-Molotov Pact with Nazi Germany only because Poland’s aggressive foreign policy (Article 33, Article 34, Article 35, Article 36, Article 37, Table 2). Moreover, Stalin is presented as a liberator and “the results of the territory occupation are intentionally omitted” (Article 38, Table 2). According to EUvsDisinfo specialists, “this case represents a manipulation of historical facts to downplay and justify Soviet aggression towards Poland, Finland, Baltic States and Romania” ( Teodor & Teodor, 2021, 178).

*6. Negating the crimes of Soviet Army and Soviet occupation in Central and Eastern Europe by promoting USSR/ Russia as a peace-maker and liberator, as a victim of Russophobia, as a victim of violations of the international law, as a victim of propaganda.*

The disinformation cases contain historical revisionism, or recurring pro-Kremlin disinformation narratives, accusations of Russo-phobia, such as in the case of the events from 13 ^th^ of January 1991 in Lithuania, in the case of Stalin’s repressions in 1937–1938, or in the case of promoting the old imperial idea that the Belarusian and Ukrainian languages are simply the “Russian” language. The pro-Kremlin policy of historical revisionism seeks to glorify the Soviet victory in World War II and downplay and relativize the crimes against humanity perpetrated by Stalin and by Soviet totalitarianism. The claim involves deceptive moral equivocation to suggest that Stalin’s „defeat” of Nazism nullifies his other crimes against humanity.

When justifying Soviet actions, there can also be identified a pattern of identifying other aggressors, such as the: US presence in Europe, portraying Europe as a vassal of the USA and trying to undermine the sovereignty of European countries” (Article 39, Article 40, Table 2). Hitler’s killing was also used to distract the killing of the communist regimes.

Presenting false information is another technique used to change the perception of facts, such as in the case of the Gulag, presented as a correctional facility and not as a concentration camp; another distortion of the truth can be associated with the Katyń Massacre.

When discussing the crimes of the Soviet Army, or of the Soviet occupation on Central and Eastern Europe, Russia denies its role by accusing political and toxic accusations, which are not sustained by proofs. The negative impact of the communist regimes is presented as propaganda, without relying on real data. Thus, Russia presents itself as a peacemaker and a liberator, by promoting the establishment of independent countries, denying the setup of repressive communist regimes in Central and Eastern Europe. Historical revisionism is aimed at promoting an underestimated responsibility of the USSR in the political developments of states such as Poland, Romania or Bulgaria. Pro-Kremlin disinformation, such as in the case of justifying the crimes from 1937–1938, or the Warsaw Pact invasion of Czechoslovakia, is aimed at covering up the crimes committed. Russia’s intents also to justify historical actions by positioning itself as a victim and also by pointing to the so-called aggressors, such as the attack in Lithuanian from 1991, which results were put on the responsibility of the Lithuanian Parliament.

Among the key messages transmitted, we can identify actions that accuse of Russophobia, anti-Russian actions, or propaganda, in the effort of the Kremlin to whitewash and instrumentalize the history of USSR/ Russia. The main targeted audiences are Lithuania, Poland, Arabic countries, Georgia, and also Russia.

The languages used in articles which negate the crimes of the Soviet regime are Russian, Polish, Arabic, Georgian and the main media outlets are represented by RT and Sputnik. Moreover, the messages are being transmitted through websites that are addressed at national level in Lithuania, or Poland, but with the domain in Russia, such as lt.sputniknews.ru, or pl.sputniknews.com, sputnik-georgia.com, cont.ws, it.sputniknews.com. Russian websites are also used, such as tvzvezda.ru, Rubaltic.ru, radiovesti.ru. Also, social media channels are also used to promote messages, such as RT online on Facebook, Twitter, or YouTube.

*7. War(s) in former Yugoslavia, especially the 1999 conflict in Kosovo, which culminated in the NATO air campaign against Serbia.*

The main narratives employed by Russian-speaking media regarding the war with former Yugoslavia were published either as stand-alone articles or as part of wider ‘analyses’ about geopolitical issues, which relied on the idea of the imperialist nature of the West. This is, according to Russian media outlets, especially visible in the contemporary crisis from Ukraine. This line of argument seeks less to discuss exactly what happened in Serbia and Kosovo 23 years ago, but to use this as a comparison with the contemporary frozen conflicts in Abkhazia, Ossetia, Transnistria, Crimea, and Donbas. The main argument that Russian media employs is that there are significant similarities between the cases of Kosovo and the break-away regions mentioned above. Thus, according to this slippery slope type of argument, if one accepts the legitimacy of the Western intervention in Kosovo, then one has to accept the legitimacy of Russian intervention in the post-soviet conflicts. Alternatively, if one rejects the justifications Russia offers, one should also reject the legitimacy of Western intervention in Kosovo.

Three lines of disinformation have been employed by the Russian media concerning the case of the 1999 bombing of Yugoslavia. The number of victims of the air raids is disputed, the intentions of NATO are questioned, and the legality of the interventions is discussed. While the last remains debatable, the first two are intentionally, deliberately misrepresented.

Concerning the number of victims of the bombing, a total of 759 dead have been directly attributed by the Humanitarian Law Center to the bombing (275 in Serbia excluding Kosovo and 484 in Kosovo), out of which there were only about 500 civilians. This is considerably lower than the 2,500 claimed by Russian media (Article 41, Table 2).

According to Human Rights Watch, there was no evidence of war crimes committed by NATO in Yugoslavia. During the NATO bombing campaign in Yugoslavia, civilians were accidentally killed in several incidents in which targets of military value were attacked. In three incidents, NATO targeted locations that were not of military value. Conversely, it could be proven that military decision-makers took extreme precautions to avoid killing civilians, carefully vetting each target and evaluating which munitions should be used ( HRW, 2000). While, according to Human Rights Watch, more care could have been taken (such as updating maps), NATO took deliberate precautions in order to avoid unnecessary civilian casualties. In some cases, civilian casualties were caused because the Yugoslav army used them as ‘human shields’ ( HRW, 2000).

The second line of revisionist approaches to the NATO air campaign in Yugoslavia concerns its motivations. According to this view, which relies on a statement by former Yugoslav president Slobodan Milosevic, the motivation of the campaign was not to stop human rights abuses by the Federal Yugoslav Government. According to Milosevic, the campaign was meant to split Yugoslavia, destroy it materially and thus eliminate a powerful actor in the Balkans which could pose a challenge to the West. This would pave the way for a new colonialism and an exploitation of the country's natural resources ( slobodan-milosevic.org, 2002).

However, Russian disinformation also adopts another perspective, strongly linked with the Russian narrative of discrimination of Russian-speaking minorities in neighbouring states. It accepts that the NATO intervention in Serbia was carried out with the aim of protecting an ethnic group that was being discriminated. However, the argument engages in a comparison with an apparently similar situation: that of Russian-speaking minorities in the republics of the former Soviet Union. Thus, if one believes that it was acceptable to intervene to save the Albanians, and that the Russian speakers around Russia are subject to human rights abuses, then this would legitimate Russian intervention in its near abroad. Of course, the empirical premise, that of discrimination against Russian speakers is introduced surreptitiously in the argument.

The third line of Russian media regarding the 1999 war in Kosovo is that concerning the legality of the intervention as well as of the subsequent declaration of independence. According to Russian media, the intervention and subsequent declaration of independence by Kosovo were either illegal or, if legal, the same case could be made for Crimea. While the actual 1999 bombing campaign was carried out without UN authorization, there were a number of previous UN resolutions condemning Serbian policies of ethnic cleansing in Kosovo. Further, the massacre of Racak involved the killing of dozens of Albanian civilians by Yugoslav forces, amounting to the beginning of a genocide. Further, before the actual attack, several attempts towards a negotiated solution were made, including the Rambouillet conference. Finally, Kosovo's declaration of independence took place 10 years after the war, after numerous negotiations. In the case of Crimea, 20 days passed from revolt to annexation.

*In what situations? Are there specific historical events mentioned in hostile narratives and offering a distorted vision of historical facts and events?*

The narratives include specific historical events, such as the events of 13 ^th^ of January 1991 in Lithuania, in regard to which is indicated the lack of proof that the Soviet soldiers clashed with civil people. On March 11 ^th^ 1990, Lithuania restored its independence of state, while Russia used political and economic pressure to maintain the republic as part of the USSR. In January 2021, Sputnik news presented the Lithuanian struggle for independence from the Soviet Union as the first ‘color revolution’ and a manoeuvre of the US Central Intelligence Agency (CIA). The broad disinformation campaign is linked to the commemoration of events when thousands of Lithuanians gathered at the TV tower, the radio and television headquarters, and the Parliament building in the capital, Vilnius, to take a stand against Soviet troops deployed to crush the Lithuanian independence.

Aiming to promote Russia as a peacemaker, in the context of World War II, Stalin’s actions are described as liberating, aiming to ensure independency for states such as Hungary, Czechoslovakia, Romania and Poland. In fact, the aforementioned states were kept under a communist regime, without the possibilities of taking any political, economic, or military decisions. In the same context of promoting Russia as a peacemaker, the lack of interference of the Soviets is presented as an act of respect for the Polish sovereignty when the martial law was introduced. Also, the Bolshevik ‘red expansionism’ was presented as a response to Poland’s initiative to start the war.

Part of the narratives situate Russia as a victim, such as the case of Stalin’s repressions in 1937–1938, which were justified through the actions of the people, who were themselves presented as aggressors in 1933–1934.

Russia justifies the invasion of Czechoslovakia through the Warsaw pact as a wall in front of the West, an action which resulted into more than 20 years of peace for the allies. In fact, the invasion aimed at ending the country’s political liberalization.

One of the distorted historical events is the example of Georgia, which, according to myvideo.ge, it joined the Soviet Union in 1921 on its own initiative. In fact, Georgia was a sovereign republic by 1921, when it was occupied by the USSR after the February-March 1921 war.

The 80 ^th^ ‘anniversary’ of the Molotov-Ribbentrop pact (23 August 1939) and Poland’s decision not to invite the Russian delegation to the World War II commemoration ceremony were the pretexts for the beginning of the fake history campaign ( Teodor & Teodor, 2021). In 2019, Poland decided not to invite Russia to the World War II commemoration ceremony, having to do with Russian aggression against Ukraine, being accused of promoting an “open policy of Russophobia” (Article 42, Table 2). On that day, in contrast, Russian propaganda stressed that in 2019 is the 80 ^th^ anniversary of the Molotov-Ribbentrop pact. That is why, Estonia, Latvia, Lithuania, Poland, and Romania released a joint statement on the 80 ^th^ commemoration of the Molotov-Ribbentrop pact, urging “the governments of all European countries to provide both moral and material support to the ongoing historical investigation of the totalitarian regimes. By acting in a concerted manner, we can counter more effectively disinformation campaigns and attempts to manipulate historical facts.” ( *Joint Statement*, 23 August 2019).

Moreover, in December 2019, Russia accused the European Parliament of trying to distort the historical truth in connection to the adoption of the resolution on the “importance of European remembrance for the future of Europe” (European Parliament, 19.09.2019). Russia expressed outrage that the resolution made the Soviet Union virtually jointly responsible with Nazi Germany for the unleashing of the Second World War.

Furthermore, after the speech of NATO Secretary General Jens Stoltenberg on the 30 ^th^ anniversary of the fall of the Berlin Wall (November 2019), the Pro-Kremlin media (Pervyi Kanal) were outraged by Stoltenberg’s phrase that it was democracy and freedom of thought that helped reunite Europe. At the Berlin Wall Memorial at NATO Headquarters, Stoltenberg emphasised that even then, Europeans were united by basic values: “freedom, democracy and human dignity” ( Stoltenberg, 2019) thanks to these, “peace and solidarity can prevail over any adversary.” ( Stoltenberg, 2019) It was freedom that was able to prevail over oppression. These shared values “allowed Europe to develop deep partnerships with friends around the world,” (Article 43, Table 2) leading to the fall of dictatorship and the spread of democracy, Stoltenberg said.

*Using what strategies (key messages and channels)?*

The Russian disinformation campaigns are adapted to every country audience specificity ( Teodor & Teodor, 2021). Disinformation and historical revisionism accusations are spread abroad overtly through their own official state-controlled externally media channels which are operational in several European languages (i.e., the multilingual television RT or Sputnik); or covertly through “independent journalists, experts and commentators […] as well as Internet trolls” ( Lucas & Pomerantsev, 2016, p. 5).

Sputnik is a Russian state-funded media organization aimed at audiences outside Russia. However, the Russian government maintains that Sputnik operates as an independent news outlet, with “its own editorial policy” (Report *Kremlin media-funded*, January 2022). Moreover, Russian government officials and the outlets’ leadership have openly discussed RT and Sputnik’s role as tools of state propaganda ( EUvsDisinfo, 2022). In order to understand the present state of the Russian media, we must look back to the Soviet era, when media was not profit-driven and the rewards were not identified in terms of revenues but by creating influential propaganda. Furthermore, the Soviet media was integrated into the Party’s control systems manifested by hiring loyalists in all key appointments, like ‘soldiers’ trained in politics and Marxist theory; using numerous of directives and instructions to editors issued by Department of Propaganda and Agitation (the ‘handouts’ which were supposed to assign which stories to be included in media outlets); reviewing and retaining media under constant scrutiny.

From the 408 disinformation cases, 200 were identified by EUvsDisinfo on Sputnik and its local versions from Poland, Germany, Serbia, Estonia, Armenia, Lithuania, Italy, Spain, Greece, Gerogia, Abkhazia or Azerbaijan and 14 cases were identified on RT and its versions in English, French or Germans ( Teodor & Teodor, 2021).

Moreover, the pro-Kremlin disinformation mechanism includes besides traditional media, television and radio programming, printed media such as periodicals, online media with all internet-hosted platforms (e.g., websites, blogs, YouTube channels etc.) where information in the form of text, video, audio, or images can be transmitted or accessed. Experts state that both RT and Sputnik operate websites and YouTube channels, and maintain a social media presence, with RT also broadcasting on traditional media (TV). RT operates several YouTube channels in different languages: RT (in Russian), RT Deutsch, RT Arabic, RT France, RT Español, RT UK, and RT America. (Matthews *et al.,* p. 37)

From the 408 disinformation cases, 47 were identified by EUvsDisinfo staff on different YouTube channels such as: Rossiya 24 – YouTube, Pervyy kanal – YouTube, RT France - YouTube, 60 minut @Rossiya 1 – YouTube, Ahí les Va – YouTube, NTV – YouTube, Dobrov v efire @REN TV – YouTube, TV Center – YouTube, OTR TV – YouTube, Petr Tolstoy Vremya Pokazhet – YouTube, RUS BG – YouTube; etc.

The social media platforms are a distinct third category. Twitter and Facebook seem to be prominent in the use of pro-Kremlin disinformation mechanisms.

Another powerful strategy consists of invoking official documents and local (Soviet) classified reports about the West or Poland planning aggressive actions against URSS right before the WWII. Moreover, conspiracy theory was used as part of the historical revisionism policy.

Recurrent narratives are also used, asserting misleading information, as in the case of accusing Western Europeans of not knowing the history of their country (e.g., France), because of poor education, ignorance and guilt. It is also the case of presenting the construction of the Berlin Wall as a desire of the Western powers, when, in fact, the Communist East German authorities built the Berlin Wall in 1961 to stop the flow of residents into the democratic, and free West; western powers and NATO allies opposed the wall from the beginning.

*With what intentions? With what assets? Producing what kind of estimated effects?*

Russia uses both visual and discursive propaganda by spreading distorted and fake messages through different state media channels in order to achieve the country’s objective. Thus, to mobilize domestic Russian audiences, the pro-Kremlin media repeatedly invokes World War II imagery and attempts to portray Lithuania, and other Baltic States as well as Poland, as anti-Semitic, Nazi and neo-fascist countries. The accusation of Nazism is one of the favourite techniques of pro-Kremlin outlets, but it is not new.

Russia’s government-funded and directed outlets are effectively influencing viewers’ political convictions ( U.S. Department of State, Special Report, 2022). RT and Sputnik’s audience reach is difficult to measure in part because RT has reportedly inflated its broadcast statistics in the past, but also because both outlets operate as part of a network composed of numerous brands, websites, and social media accounts publishing content in many languages ( U.S. Department of State, Special Report, 2022).

*What are the main targeted audiences by these narratives and why?*

We can identify likely audiences Russia and its agents seek to reach and influence on the basis of the content, as well as the ultimate reach or resonance of a given information effort.

As Keir Giles argues, Russia’s information efforts either seek to influence foreign decision-making by manipulating the information that reaches the decision makers themselves or seek to “create a permissive public opinion environment where Russian narratives are presented as factual,” and “to win public support in adversary nations, and, thereby, attenuate resistance to actions planned by Russia.” ( Giles, 2016, p. 22). Thus, at a high level of generality, audiences targeted by Russia’s malign information efforts in European countries consist of the general public, decision makers, and influencers (i.e., actors who may not be decision makers themselves but have the ability to influence decisions and public opinion).

While some malign information efforts are aimed at the general public of European countries, others appear to target public that are more narrowly defined: notably, public on the extreme political right and extreme left, especially those identified with anti-Western, anti-establishment, anti-immigrant, and nationalist viewpoints; Russian-speaking communities within former Soviet republics as well as other European states; and public with cultural, historical, religious, or political affinities to Russia, such as Slavs, the Christian Orthodox, and historical allies or partners long supported by Russia, as well as the socially conservative and otherwise pro-Russian constituencies. ( Matthews *et al.,* 2016, p. 37–38)

RT and Sputnik do not work in a vacuum within the disinformation and propaganda ecosystem. Each pillar has the potential to create a piece of disinformation or a narrative that the other pillars pick up, modify, and amplify. These relationships have a media multiplier effect that boosts each pillar’s reach and resonance. The media multiplier effect can create disinformation storms with potentially dangerous effects.

## Discussion and conclusion

Manipulative revisionist narratives, as a tool in the landscape of hybrid threats, challenge democratic societies and require knowledge and skills from a set of practitioners and stakeholders to be addressed. This suggests the need to count with expert historians in fact-checking/debuking teams to combat misinformation based on historical revisionism, due to the high specialization and complexity of historical events and their implications. For example, a journalist with poor knowledge on the history of the Central and Eastern European countries, Nordic countries, Baltic countries, or other regions, will have serious difficulties for fast responding to information manipulations with this regard. Furthermore, this type of information manipulations and narratives are easy to ‘buy’ by vulnerable groups. Universities and educational institutions play an important role in building societal resilience through education and training programmes.

Our findings suggest that an approach consisting on pre-emptively elaborating counter-narratives based on historical evidence and sound historiography can be an effective tool against hostile revisionist narratives that exploit vulnerabilities and specific target groups within European societies. Fact-checks and pre-bunks on revisionist disinformation are helpful in countering this form of interference, but their reach to non-specialized audiences is usually not high.

For dealing with narratives that aim to sow hostility towards other EU member states or alliances, it is also very important to ensure a high standard of knowledge and appreciation of history within societies, for which education at various levels is key.

With this in mind, curricula can be adapted or strengthened to teach critical thinking skills to high school students, particularly regarding the differences between cognitive values and evidence-based historiography, and historical revisionism and partisan interpretations of history. History courses provide a good opportunity to teach knowledge and skills (awareness of scientific methods, sound reasoning vs. fallacies) that can be used in other areas of knowledge and against information manipulation.

The use of historical revisionism by foreign state actors for purposes of interference also suggests that publishers must commit to high standards of academic rigor and clearly distinguish between historical essays based on authors' opinions and sound historiographical works. Institutional support for publishing to promote scholarly works on European history aimed at older and young readers could be helpful in this regard.

In this sense, it would be worth considering using the possibilities of interactive live streaming platforms, especially for young adults, to disseminate historical content with a European focus. 

## Data Availability

Zenodo: Collection of narratives gathered by academia, governments, and think-tanks. https://doi.org/10.5281/zenodo.7943019. ( Arribas *et al.,* 2023a), The project contains the following underlying data: CEE PRev DB.xlsx. (Examples of Russian revisionist narratives). Data are available under the terms of the Creative Commons Attribution 4.0 International license (CC-BY 4.0). Zenodo: Historical Revisionism Searchs. https://doi.org/10.5281/zenodo.7943014. ( Arribas *et al.,* 2023b), The project contains the following extended data: historical revisionism searchs.xlsx. (Search strategy used in this study). Data are available under the terms of the Creative Commons Attribution 4.0 International license (CC-BY 4.0).
