# Supplementary material for: Information manipulation and historical revisionism: Russian disinformation and foreign interference through manipulated history-based narratives
Source: Open Res Eur. 2023 Jul 27;3:121. [Version 1] doi: 10.12688/openreseurope.16087.1 (PMC10509605; doi:10.12688/openreseurope.16087.1)
Supplement: Peer review history

# Reviewer report: Reviewer response for version 1

**DOI:** 10.21956/openreseurope.17370.r34723

**Published:** 2023-09-19

**Authors:** Andriana Benčić Kužnar

**Affiliations:** 1 Faculty of Humanities and Social Sciences, Department of Sociology, University of Zagreb, Zagreb, Croatia

I would definitely suggest that this article be indexed concerning the very relevant and contemporary topics it employs. Nevertheless, certain aspects would need to be seriously improved before indexing.

 At the theoretical level, although authors show skillful knowledge of the term historical revisionism (p. 3–4), I would suggest better conceptualization of the term historical revisionism where it would be primarily recognized, when we conditionally speak of "bad historical revisionism" as historical denialism or negationism, which then leads to historical manipulations. From my perspective, as of now, it is not clear enough. Furthermore, many complex overlapping concepts are used without enough clarification, meaning operationalization and conceptualization (for instance, historical revisionism, historical rewriting, manipulated narratives, and propaganda), which also led to a rather complex title of this article, which can also be simplified and slightly changed if better conceptualizations are shortly introduced at the theoretical level and in the introduction. This problem is also visible at the end of the introduction, where the research goal is emphasized as examining historical revisionism-based information and influencing campaigns. I would therefore suggest the operationalization of historical revisionism before applying it to Russian foreign policy and hybrid warfare as primarily historical negationism or denialism (which is employed for manipulation purposes, although historical manipulation can also be distinguished from historical revisionism) or historical distortion. On the other hand, as the authors explain, historical revisionism also denotes a positive, fairly needed, irrefutable, and unstoppable academic revision of these same major historical events. Tucker's distinction (p. 3–4) might not be the best choice for explaining that, given the multiplicity of terms discussed later through methodological research, it is complex in itself, where terms such as disinformation, manipulation, distortion, denying, etc. are introduced without fairly explained theoretical explications.

 Authors should decide if slight changes to the title are also needed given the above discussion.

 Conceptually, narratives should be replaced with the concept of storylines (p. 4, State efforts to reconstruct history need narratives...").

 On p. 5, I would not use the syntagm "hot historical revisionist topic" or get rid of hot in this context.

 On the methodological level, it is worth thinking about whether mixed methodology is really employed. From my view, quantitative methods are not employed here directly; all methods should fall under the content analysis umbrella, and thus multiple methods are employed, not mixed methodology.

 Although the study design is really well imagined, the main discussions that follow the employed methods are too complex and too long; maybe they should be better summarized in a way to stay focused on the most important and most grounded results.

 Although the goal of this article is clear to me, and I accepted a review because of its relevance and importance in contemporary social sciences and humanities, we cannot disagree with or erase some "historical facts", where we can question Russian's further (dis)interpretations and manipulations (and commemorative events), yet we cannot state that Soviet troops have not liberated Auschwitz and that the Soviet Union at that time did not stand on the "right side of history".

 The discussion and conclusion are too short given the multiple aims set forth in this article as well as the long research analysis. Further, although it is certainly correct that universities and educational institutions play a crucial role in building societal resilience, this is not something that comes from this article.

 Ultimately, given the excellence of the authors and the potential of this article (which might be cited widely and across the globe), I certainly recommend its indexing Yet, improvements, which might seem more complex and longer than they really are, are also recommended and needed before indexing.

 The article also requires careful proofreading.

If applicable, is the statistical analysis and its interpretation appropriate?

Partly

Is the study design appropriate and is the work technically sound?

Yes

Is the work clearly and accurately presented and does it engage with the current literature?

Partly

Are the conclusions drawn adequately supported by the results?

Partly

Are sufficient details of methods and analysis provided to allow replication by others?

Partly

Are all the source data and materials underlying the results available?

Partly

Reviewer Expertise:

Collective memory, politics of memory, sociology of war and violence, Holocaust and genocide studies

I confirm that I have read this submission and believe that I have an appropriate level of expertise to confirm that it is of an acceptable scientific standard, however I have significant reservations, as outlined above.

# Reviewer report: Reviewer response for version 1

**DOI:** 10.21956/openreseurope.17370.r34722

**Published:** 2023-09-11

**Authors:** Ondrej Filipec

**Affiliations:** 1 Department of Politics and Social Sciences, Palacky University Olomouc, Olomouc, Czech Republic

In the context of the Russian full-scale invasion of Ukraine, and Kremlin revisionist (imperial) aspirations in general, it is necessary to analyse the contemporary nature and effects of historical revisionism: the Russian approach to relatively recent historical events, which are purposefully and systematically reinterpreted in the context of disinformation and propaganda, and later in practice used for justifying the aggression and clash with the West. It is a very complex issue that might be traced back to the early stages of Soviet existence and its interactions with neighbours, a critical element of the build-up and construction of the Soviet (later Russian) identity and perception of others, including Ukrainians. To some degree, Russian historical revisionism and crooked view of reality are significantly responsible for the collision course with the West and the bloody conflict in Ukraine. For that reason, I was happy, to read the article by the collective of authors who succeeded in raising the issue and highlighting some important points.

 The article has some strong and weak elements which are discussed below.

 First, next to the importance of the topic, the strong side is also a focus on contemporary historical revisionism as communicated in the individual states of Central and Eastern Europe, which is demonstrated by the evidence derived from research articles and the EUvsDisinfo database. I found both parts of the research well-fitting and complementary, despite the impression that systematic analysis of the articles led to omitting or at least underplaying some important narratives present in the individual member states. For example, this might be well developed in conversations with national experts. For instance, in the case of Czechoslovakia, a very frequent narrative pursued by the pro-Kremlin propagandist environment is that “Prague was liberated by Soviets” (which is not fully true, as Nazi resistance was eliminated during the Prague uprising and at the times of the German capitulation the Red army was far away from Prague). Similarly, a very persistent revisionist argument is that “Czechs betrayed Russia and are ungrateful for “liberation”). Both arguments were raised when Prague decided to remove the controversial Ivan Konev memorial. In other words, the national context might be better emphasized in the case of all mentioned countries maybe as evidence of how general narratives are applied in a specific national environment.  

 Second, sometimes the article is too heavy to read because of the structure, which might be simplified. For example, the questions on pages 17-19 may constitute an individual chapter, which fits well into the research design. Also, it will help the reader when each chapter has a sort of introduction, presenting the aims and structure of the chapter + a short semi-conclusion at the end presenting the most important things.

 Third, analysing the EUvsDisinfo database is an interesting and relevant approach. However, the article has some inherent limits as historical revisionism is communicated via multiple channels and the EU database reflects only a fragment of this communication. Of course, it is out of any research to make a complex assessment of the whole information ecosystem and to some degree it is even impossible (as, for example, revisionist content and interpretations are communicated in forwarded chain emails among seniors which is hard to obtain) and the very problem sometimes lies in the institutions of the target country. This is the case of the Soviet-era education system inheritance (older generations thought and accepted revisionist propaganda) and in some countries, this problem of weak history education is a never-ending story. As a result, the so-called “Overt window” is opening people's minds to revisionist interpretations that neglect historical facts.

 Fourth, despite the authors providing the broader context of revisionism, it would be interesting to develop a paragraph about the importance of historic revisionism for understanding the context of the confrontation between East and West during the Cold War, and between Russia and the West. It is worth stressing that revisionist views were developed in the West (for example, Williams 1962 ^1^ and 1974 ^2^, Kolko and Kolko 1972 ^3^; Yergin 1977 ^4^ or even post-revisionists like John Lewis Gaddis, 1987 ^5^).

 Fifth, I agree that measures proposed in the discussion and conclusion are significantly helpful in addressing Russian revisionist aspirations, however, it is also time for the adjustment of government policies, effective strategic communication, or supporting assemblage of the civil society. In the measures, proposed, authors might provide references on resources developing the ways why and how these measures might help.

 Sixth, many authors are dealing with Russian disinformation and propaganda as a part of Russian revisionists. Authors might consider exploring works, for example of Yablokov and others (Yablokov 2018 ^6^, Verovšek 2016 ^7^, Topolski 1999 ^8^, Juuervee *et al.* 2020 ^9^; Prus 2015 ^10^). By adding more references, the contribution will be more visible on academic social networks, attract more attention, and increase the context of academic research.

 The above comments are not critical for the quality of the manuscript, which fully matches the scientific text's characteristics. I wish the authors all the best with the publication.

If applicable, is the statistical analysis and its interpretation appropriate?

Not applicable

Is the study design appropriate and is the work technically sound?

Yes

Is the work clearly and accurately presented and does it engage with the current literature?

Partly

Are the conclusions drawn adequately supported by the results?

Yes

Are sufficient details of methods and analysis provided to allow replication by others?

Yes

Are all the source data and materials underlying the results available?

Yes

Reviewer Expertise:

European Union, Hybrid Threats, Hybrid Warfare, Disinformation and Propaganda

I confirm that I have read this submission and believe that I have an appropriate level of expertise to confirm that it is of an acceptable scientific standard.

## References

[1] : American Innocence Questioned, In: T. Paterson (ed.), The Origins of the Cold War, 2nd edition. *Oxford:Oxford University Press* .1974;225-229

[2] : The Tragedy of American Diplomacy. *New York: Norton* .1962;

[3] : The Limits of Power: The World and United States Foreign Policy, 1945-1954. *New York: Harper and Row* .1972;

[4] : Shattered peace: The origins of the Cold War and the National Security State. *Boston: Houghton Mifflin.* .1977;

[5] : The Long Peace. Inquiries into the History of the Cold War. *Oxford: Oxford University Press* .1987;

[6] : Fortress Russia: Conspiracy theories in the post-Soviet world. *Polity: Cambridge and Medford* .2018;

[7] : Collective memory, politics, and the influence of the past: the politics of memory as a research paradigm. *Politics, Groups, and Identities* .2016;4(3) : 10.1080/21565503.2016.1167094 529-543 10.1080/21565503.2016.1167094

[8] : The Role of Logic and Aesthetics in Constructing Narrative Wholes in Historiography. *History and Theory* .1999;38(2) : 10.1111/0018-2656.00086 198-210 10.1111/0018-2656.00086

[9] : Falsification of history as a source of influence. Centre. *Riga: NATO Strategic Communications* .2021; Reference source

[10] : Russia's use of history as a political weapon. *The Polish Institute of International Affairs* .2015; Reference source

# Reviewer report: Reviewer response for version 1

**DOI:** 10.21956/openreseurope.17370.r34125

**Published:** 2023-08-16

**Authors:** Elias Said-Hung

**Affiliations:** 1 International University of La Rioja, Logroño, La Rioja, Spain

I consider it a very interesting and valuable work, although I also consider that it needs some aspects of improvement to achieve the relevance that I think it should have. Here are some aspects of improvement:

- At the level of literary review: Current works should be reviewed that highlight the role of disinformation content in conditioning public opinion by Russia. Many reports (Cambridge Analytical) and authors (e.g. Ablazov et al., 2021; Bodrunova et al., 2021). Furthermore, I believe there is a lack of connection between the literature and the role of journalism, the training of professionals in the sector, and the role of fact-check projects. I point out the latter because it is where the discussions and conclusions raised the point.
- At the methodology level: It would be convenient to review the proposed general objective to make it more specific. Also, the reasons for selecting EUvsDisinfo should be more explicit and evident. In addition, the corpus of analysis considered during the proposed documentary review should also be made more explicit as its relationship with the literary review carried out. The latter is to have a clear association between the proposed theoretical framework and how the contents found and analyzed in the EUvsDisinfo are analyzed.
- The search strategy used by Arribas et al., 2023b is alluded to, but it should be explained or shown through some figure as a summary within the work. This is in order to facilitate the understanding of the applied process.
- In principle, the crossing of data is considered. On the one hand, those found in EUvsDisinfo, and on the other, those found in academic databases. And also from research papers and reports from Baltic countries. The search process in each medium considered should be explained more clearly, justifying the selection of said sources and the combination and operators used to search for information required for the development of this work. It should also justify why October 2021 was taken as the sampling date. It would be helpful to clarify the association between the types of data extracted and the specific objectives set.
- At the level of the results, it is considered that this section should be better reorganized, making the above more evident concerning each of the proposed objectives. Beyond the structure proposed for its exposition, this does not seem wrong but leaves the reader with the need to establish this association based on the results shown.
- At the level of discussion and conclusions. It should be reviewed. Perhaps it would be convenient to divide both sections in order to have, on the one hand, the reflections associated with the data and the proposed theoretical framework; and, on the other, the limitations, future lines and practices of the knowledge generated. Although what is indicated in this section seems interesting to me, what is exposed is disassociated with the literature review and data types. Considering that what these authors propose is very useful and relevant, the association of the rest of the sections with what they present here is unclear.

If applicable, is the statistical analysis and its interpretation appropriate?

Partly

Is the study design appropriate and is the work technically sound?

Yes

Is the work clearly and accurately presented and does it engage with the current literature?

Partly

Are the conclusions drawn adequately supported by the results?

Partly

Are sufficient details of methods and analysis provided to allow replication by others?

Partly

Are all the source data and materials underlying the results available?

Yes

Reviewer Expertise:

Social media, Disinformation content and Hate Speech.

I confirm that I have read this submission and believe that I have an appropriate level of expertise to confirm that it is of an acceptable scientific standard, however I have significant reservations, as outlined above.

## References

[1] : Report on disinformation. Reference source

[2] GEC Special Report: Russia’s Pillars of Disinformation and Propaganda. Reference source

[3] : Disinformation and Russia’s war of aggression against Ukraine: Threats and governance responses. Reference source

[4] REPORT on foreign interference in all democratic processes in the European Union, including disinformation. Reference source

[5] RUSSIAN DISINFORMATION ATTACKS ONELECTIONS: LESSONS FROM EUROPE. Reference source

[6] Countering disinformation with facts - Russian invasion of Ukraine. Reference source

[7] ANALYSIS OF RUSSIA’S INFORMATION CAMPAIGN AGAINST UKRAINE. Reference source

[8] : Countering disinformation for the promotion and protection of human rights and fundamental freedoms. Reference source

[9] : Disinformation as a form of aggression: Ukraine and its partners amidst the Russian fake news (early 2021).2021;65-72 10.5281/ZENODO.4959700

[10] : Disinformation as a Threat to Deliberative Democracy. *Political Research Quarterly* .2021;74(3) : 10.1177/1065912920938143 703-717 10.1177/1065912920938143

[11] : Assembling the Networks and Audiences of Disinformation: How Successful Russian IRA Twitter Accounts Built Their Followings, 2015–2017. *Journal of Communication* .2021;71(2) : 10.1093/joc/jqaa042 305-331 10.1093/joc/jqaa042

[12] : Constructive Aggression? Multiple Roles of Aggressive Content in Political Discourse on Russian YouTube. *Media and Communication* .2021;9(1) : 10.17645/mac.v9i1.3469 181-194 10.17645/mac.v9i1.3469
